# *Candida albicans* Carriage in Children with Severe Early Childhood Caries (S-ECC) and Maternal Relatedness

**DOI:** 10.1371/journal.pone.0164242

**Published:** 2016-10-14

**Authors:** Jin Xiao, Yonghwi Moon, Lihua Li, Elena Rustchenko, Hironao Wakabayashi, Xiaoyi Zhao, Changyong Feng, Steven R. Gill, Sean McLaren, Hans Malmstrom, Yanfang Ren, Robert Quivey, Hyun Koo, Dorota T. Kopycka-Kedzierawski

**Affiliations:** 1 Eastman Institute for Oral Health, University of Rochester Medical Center, Rochester, NY, United States of America; 2 Department of Dentistry, North Sichuan Medical University, Sichuan, China; 3 Department of Biochemistry and Biophysics, University of Rochester Medical Center, Rochester, NY, United States of America; 4 School of Dentistry, Peking University, Beijing, China; 5 Department of Biostatistics and Computational Biology, University of Rochester Medical Center, Rochester, NY, United States of America; 6 Genomics Research Center, University of Rochester Medical Center, Rochester, NY, United States of America; 7 Department of Orthodontics and Pediatric Dentistry & Community Oral Health Divisions, School of Dental Medicine, University of Pennsylvania, Philadelphia, PA, United States of America; University of Florida, UNITED STATES

## Abstract

**Introduction:**

*Candida albicans* has been detected together with *Streptococcus mutans* in high numbers in plaque-biofilm from children with early childhood caries (ECC). The goal of this study was to examine the *C*. *albicans* carriage in children with severe early childhood caries (S-ECC) and the maternal relatedness.

**Methods:**

Subjects in this pilot cross-sectional study were recruited based on a convenient sample. DMFT(S)/*dmft(s)* caries and plaque scores were assessed during a comprehensive oral exam. Social-demographic and related background information was collected through a questionnaire. Saliva and plaque sample from all children and mother subjects were collected. *C*. *albicans* were isolated by BBL^™^ CHROMagar^™^ and also identified using germ tube test. *S*. *mutans* was isolated using Mitis Salivarius with Bacitracin selective medium and identified by colony morphology. Genetic relatedness was examined using restriction endonuclease analysis of the *C*. *albicans* genome using BssHII (REAG-B). Multilocus sequence typing was used to examine the clustering information of isolated *C*. *albicans*. Spot assay was performed to examine the *C*. *albicans* Caspofungin susceptibility between S-ECC children and their mothers. All statistical analyses (power analysis for sample size, Spearman’s correlation coefficient and multiple regression analyses) were implemented with SAS 9.4

**Results:**

A total of 18 S-ECC child-mother pairs and 17 caries free child-mother pairs were enrolled in the study. Results indicated high *C*. *albicans* carriage rate in the oral cavity (saliva and plaque) of both S-ECC children and their mothers (>80%). Spearman’s correlation coefficient also indicated a significant correlation between salivary and plaque *C*. *albicans* and *S*. *mutans* carriage (p<0.01) and caries severity (p<0.05). The levels of *C*. *albicans* in the prepared saliva and plaque sample (1ml resuspension) of S-ECC children were 1.3 ± 4.5 x10^4^ cfu/ml and 1.2 ± 3.5 x10^4^ cfu/ml (~3-log higher vs. caries-free children). Among 18 child-mother pairs, >60% of them demonstrated identical *C*. *albicans* REAG-B pattern. *C*. *albicans* isolated from >65% of child-mother pairs demonstrated similar susceptibility to caspofungin in spot assay, while no caspofungin resistant strains were seen when compared with *C*. *albicans* wild-type strain SC5314. Interestingly, the regression analysis showed that factors such as antibiotic usage, birth weight, inhaler use, brushing frequency, and daycare attendance had no significant effect on the oral carriage of *C*. *albicans* in the S-ECC children.

**Conclusions:**

Our results reveal that both the child with S-ECC and the mother were highly infected with *C*. *albicans*, while most of the strains were genetically related, suggesting that the mother might be a source for *C*. *albicans* acquisition in the oral cavity of children affected by the disease.

## Introduction

Early childhood caries (ECC) is the single most common chronic childhood disease and is known to disproportionately afflict up to 72.7% of underprivileged preschool children in both developing and industrialized countries [1]. ECC is defined as “the presence of one or more decayed (non-cavitated or cavitated lesions), missing teeth (due to caries), or filled tooth surfaces in any primary tooth in a child 72 of months age or younger [2]. In children younger than 3 years of age, any sign of smooth-surface caries is indicative of severe early childhood caries (S-ECC) [2]. From ages 3 through 5, one or more cavitated, missing teeth (due to caries), or filled smooth surfaces in primary maxillary anterior teeth, or decayed, missing, or filled score of ≥4 (age 3), ≥5 (age 4), or ≥6 (age 5) surfaces constitutes S-ECC [2]. The onset and progression of ECC is rapid, aggressive, painful, recurrent, and frequently requires total oral rehabilitation under general anesthesia at towering costs [3]. Consequences of ECC include higher risk of new carious lesions, risk for delayed physical growth and development, loss of school days, and hospitalizations and emergency room visits [4]. Thus, ECC constitutes a major challenge for public health systems [5].

Dental caries is a transmissible, and biofilm-dependent infectious disease [6, 7]. Understanding the acquisition of cariogenic microbes are important for development of improved preventive strategies. Traditional microbial risk markers for ECC include *Streptococcus mutans* and *Lactobacillus* species, whose transmission routes have been thoroughly investigated [8–14]. In the past decade, the fungus *Candida albicans* has been frequently detected in high numbers together with *S*. *mutans* in oral biofilms (known as dental plaque) collected from children with ECC [15–18]. Clinical studies have provided evidence of the positive association of Candida carriage and occurrence of caries in children [14–21]. *In vitro* studies have also demonstrated an interaction between *C*. *albicans* and *S*. *mutans* in the presence of sucrose, leading to co-species biofilm development [22–27]. Furthermore, a recent *in vivo* study have shown that the presence of *C*. *albicans* enhances the formation of highly cariogenic biofilms, causing the onset of rampant caries in an animal model of the disease [23]. Although previous research suggested the cariogenic association between *C*. *albicans* and ECC, to date, the transmission patterns of *C*. *albicans* within ECC children have not been characterized.

Fungal colonization in neonates, especially in very low birth weight infants has been investigated in pediatric medicine. Both vertical and horizontal transmission were implicated. The vertical transmission rate ranges from 14% typed by electrophoretic karyotyping and restriction endonuclease analysis of genomic DNA with pulsed-field gel electrophoresis, to 41% typed by DNA fingerprinting using a *C*. *albicans* strain-specific DNA probe [28, 29]. Molecular epidemiology has been used to analyze genetic relationship and to identify the route of transmission for *C*. *albicans* [30]. Methods such as pulsed-field gel electrophoresis (PFGE), Ca3 fingerprinting, and multi-locus gene sequencing typing offer high-resolution, greater stability, and they have proved to be more discriminatory than phenotypic methods [31]. Restriction endonuclease analysis of the genome using BssHII (REAG-B) is one of the PFGE assay, and it has proven to be a reliable method with high discriminatory abilities for *C*. *albicans* relatedness research [29, 32–36]. It has been successfully used to investigate outbreaks and performed well when compared with other typing techniques [37].

Identifying the acquisition source of *C*. *albicans* to children with ECC may be an important component to help prevent the onset and relapse of this highly infectious and difficult to treat disease. The main purpose of this study was to examine the carriage of *C*. *albicans* and the maternal relatedness of *C*. *albicans* in children with S-ECC using a combination of culturing and genetic (REAG-B and multilocus sequence typing) methods. We hypothesized that the mother and the child with ECC were both infected with *C*. *albicans*, and that *C*. *albicans* strains isolated from children with S-ECC were genetically related with the *C*. *albicans* isolated from their mothers.

## Materials and Methods

### Study design

This cross-sectional study was single centered and carried out at the Eastman Institute for Oral Health (EIOH) at the University of Rochester. Ethical approval of the study and the written consent/permission forms were obtained from the Research Subject Review Board at the University of Rochester (RSRB00056870) prior to the study commencement. A convenience sample of children with S-ECC and their mothers were recruited to the study. These children were patients seen at EIOH from June 2015 to September 2015, whose oral health condition required oral rehabilitation in the operating room under general anesthesia. Study subjects provided written informed consent to participate in this study. For subjects younger than 7 years old, written permission form was reviewed and signed by their legal guardians. The sample size calculation was based on the estimated proportion of 60% that the children and mothers shared identical *C*. *albicans* strains in our pilot study, compared with the proportion of 14–41% in the general population as reported in the literature [28, 29]. The average of the reported proportions was included as the null proportion (25%) and used in a z-test with a one-sided alternative at alpha = 0.05. The needed sample size to achieve 90% power comprised of 15 child-mother pairs.

### Inclusion and exclusion criteria

The inclusion criteria for the children were: 1) 12–71 months of age; 2) diagnosis of S-ECC, defined by the 2014 Reference Manual of the American Academy of Pediatric Dentistry (AAPD); 3) at least one active untreated carious lesion; 4) mother is also willing to take part in the study. Children and mothers who received local and/or systemic antifungal therapy within 90 days of the sample collection visit were excluded from the study. As control group, caries free children aged from 12–71 months of age and their mother were also enrolled, same exclusion criteria was applied except caries lesion.

### Comprehensive oral examination

A comprehensive oral examination was performed on the day of the sample collection by one of the two-calibrated dentists, using a standard dental mirror and CPI probe, with artificial headlight. The number of teeth was determined, and dental caries status of the dentition was charted using decayed (D), missing due to decay (M), or filled (F) index according to the codes proposed by WHO Oral health surveys—basic methods, 4^th^ edition, 1997 [38]. Specifically, DMFT(S) for permanent dentition teeth and surfaces and *dmft(s)* for primary dentition teeth and surfaces. Radiographic bitewings were also used to assess the occlusal and interproximal caries, and used for decayed teeth/surfaces calculation. Plaque status for adult was examined and charted for 6 index teeth according to criteria modified from Silness and Löe [39]. Plaque status for primary dentition and mixed dentition was examined and recorded according to criteria modified from Ribeiro *et al*, 2002 [40]. Only a single score was used for the whole dentition: 0, for absence of visible plaque; 1, for thin plaque, in anterior and/or posterior teeth, visible just after drying with gauze; 2, for thick plaque, in anterior or posterior teeth, visible without drying; 3, for thick plaque, firmly adhered, in anterior and posterior teeth, visible without drying. Inter- and intra-examiner agreement for the evaluated criteria was calculated by Kappa statistics, and exceeded 82% at the calibration.

### Medical-social-demographic survey

A survey (S1 File) including questions related to medical-social-demographic background was completed by the children’s mother. Questions regarding oral hygiene behavior, previous yeast infection history, antibiotic use, birth weight, and daycare attendance were asked.

### Clinical sample collection

Subjects were asked not to brush the teeth 2 hours before sample collection. Concentrated oral rinse [41, 42] was used for salivary sample collection for mothers. Subjects were asked to rinse the mouth with 10 ml of sterile 0.9% sodium chloride irrigation solution, USP (Baxter, Deerfield, USA), for 1 min prior to the collection in a sterile 50ml centrifuge tube. The oral rinse was centrifuged at 2,000 × *g* for 10 min, the supernatant was discarded, and the deposit was resuspended in 1ml of 0.9% sodium chloride solution. The saliva sample of S-ECC children was collected through a disposable saliva ejector attached to a 15 mL sterile centrifuge tube, which in turn was attached to a vacuum pump [43, 44]. Approximately 1 ml of saliva was collected for each subject. After collection, the sample was stored on ice and transferred to the lab located at the Center for Oral Biology at the University of Rochester within 2 hours. Each saliva sample was sonicated on ice by the Branson Sonifier 150 (Branson Ultrasonics Corp., Danbury, CT, USA) to disperse the cell clumps. The swab sample was taken by rubbing the buccal mucosa and back of the tongue using a sterile cotton transport swab and transferred using the modified liquid Stuart’s transport medium (Startplex Scientific Inc, Etibicoke, Canada). Dental plaque was collected throughout the whole dentition using a sterilized periodontal scaler. The sample was suspended in 1ml of a 0.9% sodium chloride solution in a sterilized Eppendorf tube. The plaque sample was gently vortexed and sonicated (10sec sonication, 30sec rest on ice, repeat three times) to break down the aggregated plaque before plating.

### Isolation of *C*. *albicans* and *S*. *mutans*

All saliva/concentrated oral rinse, plaque, and swab samples were plated within 2 hours onto BBL^™^ CHROMagar^™^ Candida (BD, Sparks, MD, USA) and incubated at 37°C for 48 hours to isolate *C*. *albicans*. This medium permitted presumptive identification of several clinically important Candida species including *C*. *albicans* based on colony color and morphology [45]. *C*. *albicans* was also identified using germ tubing test from three randomly selected colonies in each sample. The saliva and plaque samples were plated on Mitis Salivarius with Bacitracin selective medium and incubated at 37°C for 48h to isolate *S*. *mutans* and identified by colony morphology [46].

### REAG-B typing

REAG-B typing was performed through Pulsed-Field Gel Electrophoresis (PFGE) of genomic DNA cleaved using the restriction endonuclease BssHII as described previously [33–35]. Electrophoresis was performed with a contour-clamped homogenous electric field apparatus (CHEF DR-II; Bio-Rad, California, USA). A Lambda DNA ladder (Bio-Rad California, USA) was included as the size marker. The genetic relatedness analysis was performed with the Bionumerics software (Applied Maths, Austin, TX, USA). The software assigned bands automatically. Each gel was normalized to the respective size markers (Lambda DNA ladder). The position tolerance and optimization was 3 and 3%, respectively, which was modified from the previous study by Chen et al, 2005 [47]. Dice coefficients were used to calculate the similarity percentage of the band patterns. The isolates were considered “genetically identical” when all the bands (100%) observed by the PFGE matched. Banding patterns with ≥95% but less than 100% of the bands matching were termed “genetically similar.” Isolates with less than 95% of the bands matching were considered “genetically different.”

### Multi locus sequence typing (MLST)

MLST is a DNA-based method that examines nucleotide sequence variation in seven housekeeping genes and is highly reproducible between different laboratories. MLST has a discriminatory power of 0.999 and moderate degree of easy of use [48]. MLST of *C*. *albicans* has been used widely to assess the genetic relatedness of isolates recovered from disparate geographic locations and for *C*. *albicans* population analysis [49] [50]. *C*. *albicans* strains isolated from ECC children were typed using an MLST scheme [49]. The internal regions of seven housekeeping genes (*AAT1a*, *ACC1*, *ADP1*, *MPIb*, *SYA1*, *VPS13*, *and ZWF1b*) were sequenced. Each strain was characterized by a diploid sequence type (DST) resulting from the combination of the genotypes obtained at the seven loci. The dendrogram was constructed by using the unweighed pair-group method. Sanger sequence analysis and merger was performed using CAP3. The reference database used to build the MLST scheme was the *C*. *albicans* library from PubMLST. Lineage assignment/tree construction was performed using START2.

### Spot assay

The spot dilution assay was performed on solid YPD medium with different concentration of caspofungin as described previously [51]. Briefly, cells from a −80°C stock were streaked for independent colonies on YPD plates and incubated at 37°C until young colonies of the approximate size of 1 × 10^5^ to 3 × 10^5^ cells/colony grew up. Colonies then were collected, and serial 10-fold dilutions were prepared in sterile distilled water with the aid of a hemacytometer. The corresponding suspensions were plated at 10^4^, 10^5^, 10^6^ CFU per spot on the YPD medium with 7.5ng/ml, 15ng/ml and 30ng/ml caspofungin. The plates were incubated for 5 days at 37°C and photographed with a Molecular Imager Gel Doc XR+ system (Bio-Rad laboratories, Inc. California, USA). Caspofungin inhibits the enzyme (1→3)-β-D-glucan synthase and thereby disturbs the integrity of the fungal cell wall, killing the organism. The agar-based spot assay has been reported to be a precise and reproducible method for determining relative caspofungin susceptibility [51, 52]. In our study, the rationale of performing caspofungin susceptibility testing was to examine microbiological differences between the *C*. *albicans* strains isolated from child and their mother.

### Statistical analysis

Descriptive characteristics of age, gender, plaque index, DMFT/*dmft* index and *C*. *albicans/ S*.*mutans* CFUs were analyzed. Correlation between *C*.*albicans / S*.*mutans* and caries severity was examined using Spearman’s correlation coefficient. A multiple regression analysis using the DMFT(S)/*dmft(s)* scores as the dependent variable and the genetic relatedness of *C*. *albicans*, CFUs of *C*. *albicans*, age, gender, plaque index, and medical history (yeast infection) as the independent variables was performed to explore the potential importance of *C*. *albicans* infection in S-ECC outcomes. All statistical analyses were implemented with SAS 9.4 (SAS Institute Inc., Cary, NC).

## Results

In a period of 4 months, a consecutive series of 18 S-ECC children who were scheduled to receive oral rehabilitation in operating room were enrolled. A total of 18 S-ECC child-mother pairs were recruited, which was above the needed sample size based on power analysis (Materials and Methods) for the purpose of this study. According to the mothers, most of the children in the study have not received any dental care prior to the oral rehabilitation. The demographic, social, oral hygiene, yeast infection history, was listed in Table 1. Among the 18 children with S-ECC, there were 10 boys and 8 girls; the average age was 3.9 ± 0.9 years of age. There were approximately 66% Caucasian, 17% African American, and 17% Asian children; the majority of the children (89%) were non-Hispanic. The average age of the 18 mothers was 31.8 ± 6.8 years of age. Four children and one mother had a history of yeast infections; its clinical demonstration in the children included cradle cap and diaper rash yeast infections; its form in the mother manifested with oral thrush. No subjects in the study reported long-term (>3 months) antibiotic use. Only one child was a low birth weight infant at delivery as reported by the mother. As reported in the questionnaires, two children and two mothers had a history of inhaler use for asthma. According to the mothers, 61% of the children were brushing their teeth twice/daily, 28% once daily, and 11% less than once daily. The majority of the children (78%) stayed at home with their family members and did not attend childcare programs.

**Table 1:** Social-demographic and related background information of the study subjects.

Variables		Children			Mothers		
		S-ECC (n = 18)	Caries free (n = 17)	P-value	S-ECC (n = 18)	Caries free (n = 17)	P-value
Age (year): mean ± SD		3.9±0.9	3.6±1.5	0.48	31.8±6.8	32.2±7.1	0.87
Gender: female % (n)		44% (8)	47% (8)	1.00			
Race: % (n)	Caucasian	66% (12)	41% (7)	0.28	66% (12)	41% (7)	0.29
	African American	17% (3)	41% (7)		17% (3)	35% (6)	
	Asian	17% (3)	18% (3)		17% (3)	24% (4)	
Ethnicity: Hispanic % (n)		11% (2)	18% (3)	0.66	11% (2)	18% (3)	0.66
Tooth brushing (times/day): % (n)	2	61% (11)	88% (15)	0.15	66% (12)	94% (16)	0.13
	1	28% (5)	12% (2)		28% (5)	6% (1)	
	<1	11% (2)	0% (0)		6%(1)	0% (0)	
Attending daycare: % (n)	Full time	22% (4)	29% (5)	0.13			
	Part time	0	24% (4)				
	Home care	78% (14)	47% (8)				
Yeast infection history: % (n)		22% (4)	6% (1)	0.34	6% (1)	0% (0)	1.00
Antibiotic use >3 months: % (n)		0% (0)	0% (0)		0% (0)	0% (0)	
Low birth weight: % (n)		6% (1)	12% (2)	0.60	0% (0)	6% (1)	0.49
Asthma inhaler use: % (n)		11% (2)	18% (3)	0.66	11% (2)	12% (2)	1.00

As control, 17 caries free children-mother pairs were also enrolled; the average age of the caries free children was 3.6 ± 1.5 years of age. Among them, there were 9 boys and 8 girls, 41% Caucasian, 41% African American and 18% Asian. According to the mothers, 88% of the children were brushing teeth twice/daily. In addition, 47% of the caries free children were receiving home care, which was lower than 78% in S-ECC group. One caries free child had previous yeast infection history, 2 children were born with low birth weight, 3 children were using inhaler for asthma.

### Carriage of *C*. *albicans/ S*. *mutans* and caries score

The detection rate and CFU of *C*. *albicans* and *S*. *mutans* was shown in Table 2. The *C*. *albicans* detection ranked the highest in the oral rinse and plaque samples of mothers (83.3%) and plaque samples of S-ECC children (83%). The saliva/oral-rise and plaque samples yielded higher detection rates than the swab samples. *S*. *mutans* was detected in both the saliva/oral-rinse and plaque samples in all the subjects at high detection rate (≥94%). The CFU of *C*. *albicans* in S-ECC children’s saliva and plaque was 1.3 ± 4.5 x10^4^ and 1.2 ± 3.5 x10^4^ per ml respectively. *Candida tropicalis*, *Candida Krusei* and *Candida glabrata* were also identified in 6% (1/18), 17% (3/18), and 6% (1/18) of the S-ECC children, and 6% (1/18), 28% (5/18), 6% (1/18) of the family members. The *S*. *mutans* carriage in the saliva and plaque samples of the S-ECC children was 7.6 ± 1.1 x10^6^ and 36.7 ± 46.0 x10^6^ CFU/ml respectively.

**Table 2:** Carriage of *C*. *albicans* and *S*. *mutans* and caries score in the S-ECC children and their mothers.

Variables		Children			Mothers		
		S-ECC (n = 18)	Caries free (n = 17)	P-value	S-ECC (n = 18)	Caries free (n = 17)	P-value
*C*. *albicans* (Detection)	Saliva/Oral-rinse^¶^	77%	12%	0.0005	83%	29%	0.002
	Plaque	83%	6%	<0.0001	83%	12%	0.0001
	Swab	44%	6%	0.04	50%	12%	0.03
*S*. *mutans* (Detection)	Saliva/Oral-rinse^¶^	100%	53%	0.001	94%	83%	0.66
	Plaque	100%	47%	0.0001	94%	65%	0.12
*C*. *albicans* (CFU/ml)	Saliva/Oral-rinse^¶^	1.3±4.5x10^4^	3.8±12.4	<0.0001	2.3±3.3x10^3^	2.5±5.4x10^*2*^	<0.0001
	Plaque	1.2±3.5x10^4^	4.1±17.0	<0.0001	1.7±4.1x10^3^	1.9±7.2x10^*2*^	<0.0001
	Swab	1.3±4.2x10^3^	2.9±12.1	<0.0001	0.3±0.5x10^3^	6.7±24.5	<0.0001
*S*. *mutans* (10^6^ CFU/ml)	Saliva/Oral-rinse^¶^	7.6±1.1	0.3±0.6	<0.0001	2.8±5.3	1.3±3.4	0.32
	Plaque	36.7±46.0	2.2±8.0	<0.0001	34.5±70.2	4.9±1.1	<0.0001
DT/dt		10.8±4.8	0		5.5±5.5	0.5±1.1	0.0006
MT/mt		0.1±0.5	0		2.2±2.5	0.9±2.5	0.13
FT/ft		0.3±0.8	0		2.5±3.2	2.3±2.6	0.84
DMFT/dmft		10.8±5.1	0		9.9±7.0	3.8±2.6	0.002
DS/ds		24.7±15.5	0		10.6±11.4	0.7±1.4	0.0008
MS/ms		0.6±2.3	0		10.8±12.3	4.7±12.7	0.16
FS/fs		1.1±3.6	0		4.4±5.8	3.4±4.0	0.55
DMFS/dmfs		26.4±14.3	0		25.8±22.4	8.7±13.4	0.01
Plaque index		1.9±0.7	0.8±1.0	0.0007	2.0±0.7	1.0±0.8	0.0004

^¶^ Saliva sample for children, concentrated oral rinse sample for mothers

In the caries free group, the *C*. *albicans* detection rate was significantly lower when compared with S-ECC group. *C*. *albicans* was only detected in 12% of the saliva samples, 6% of the plaque and swab samples from caries free children. *C*. *albicans* was detected in 29% of the oral rinse sample, 12% of the plaque and swab samples from caries free children’s mother. The CFU of *C*. *albicans* in caries free children’s saliva and plaque was 3.8 ± 12.4 and 4.1 ± 17.0 per ml, which was ~3 log less compared to the CFU counts found in S-ECC samples. Furthermore, *S*. *mutans* detection in caries free children and mothers was significantly lower than the S-ECC as well, the carriage in the saliva and plaque samples of the caries free children was 0.3 ± 0.6 x10^6^ and 2.2 ± 8.0 x10^6^ CFU/ml respectively, which was one log lower than the carriage in S-ECC children.

DMFT(S)/*dmft(s)* were recorded through a comprehensive oral exam, shown in Table 2. The mean *dmft and dmfs scores* for the S-ECC children were 10.8 ± 5.1 and 26.4 ± 14.3, respectively; the major portion of the *dmft(s)* in the children was comprised of decayed teeth and surfaces. The mean DMFT and DMFS scores for the S-ECC mothers were 9.9 ± 7.0 and 25.8 ± 22.4, respectively. As expected, the mothers of caries free children demonstrated significantly lower value of DT/S, DMFT/S when compared with the mothers of S-ECC children. The plaque index was 2 ± 0.7 and 1.9 ± 0.7 for the S-ECC children and mothers and 0.8 ± 1.0 and 1.0 ± 0.8 for the caries free children and mothers. Thus, the caries levels and severity as well as *C*. *albicans* carriage were both elevated in S-ECC children and the respective mothers.

The correlations between the *C*. *albicans*, *S*. *mutans*, and *dmft(s)* analyzed by Spearman’s rank correlation test were listed in Table 3. The results demonstrated a significant correlation between salivary and plaque *S*. *mutans* and *C*. *albicans* (with an index of 0.83 and 0.68, respectively; p<0.01) as well as with caries severity (p<0.05).

**Table 3:** Spearman’s rank correlation between *C*. *albicans*, *S*. *mutans and caries score among S-ECC children*.

	Saliva *Ca*	Saliva *Sm*	Plaque *Ca*	Plaque *Sm*	Swab *Ca*
Saliva *Ca*	1				
Saliva *Sm*	0.83**	1			
Plaque *Ca*	0.69**	0.68**	1		
Plaque *Sm*	0.18	0.57**	0.59**	1	
Swab *Ca*	0.61**	0.52**	0.65**	0.51**	1
*dmft*	0.11	0.51**	0.38*	0.36*	0.14
*dt*	0.20	0.57**	0.44*	0.45*	0.22
*dmfs*	0.12	0.48*	0.44*	0.25	0.15
*Ds*	0.28	0.56**	0.55**	0.29	0.21

** Correlation is significant at the 0.01 level (1-tailed)

* Correlation is significant at the 0.05 level (1-tailed)

The regression analysis showed that none of the factors such as antibiotic usage, birth weight, inhaler use, brushing frequency, and daycare attendance had significant effect on the carriage of salivary and plaque *C*. *albicans* in the S-ECC children.

### Relatedness evaluation by REAG-B and MLST

**Fig 1:**
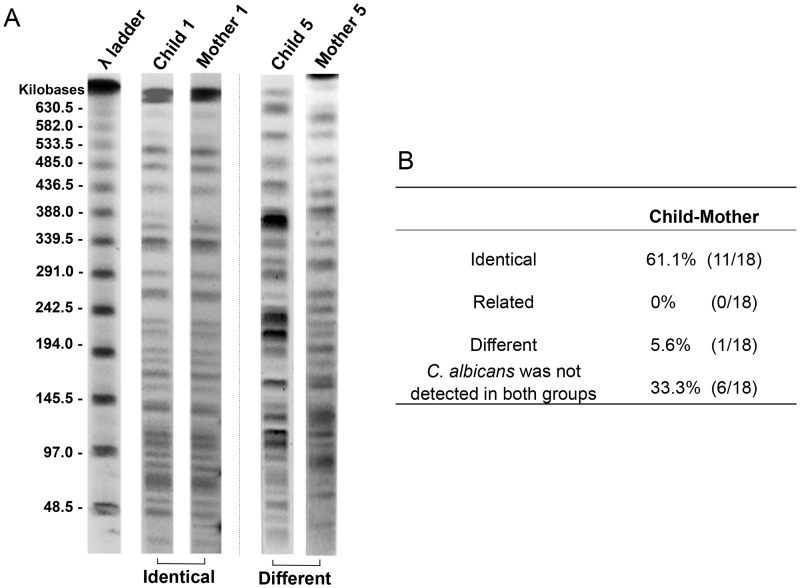
Representative PFGE patterns and genetic relatedness of *C*. *albicans* obtained by restriction endonuclease analysis of genomic DNA using BssHII (REAG-B) among family members. (A) Representative PFGE patterns and genetic relatedness of *C*. *albicans* within different child-mother pairs; (B) Relatedness of *C*. *albicans* among S-ECC child-mother pairs. * Relatedness analysis was performed by BioNumerics software (Applied Maths, Austin, TX). The *C*. *albicans* isolated from the child-1 and mother-1 were genetically identical, the *C*. *albicans* isolated from the child-5 and mother-5 were genetically different.

Having shown high fungal carriage in both S-ECC children and their mothers, REAG-B was used to exam the genetic relatedness of *C*. *albicans* isolated from each of the 18 S-ECC child-mother pairs. Among them, *C*. *albicans* was both detected in 12 pairs. In another 6 child-mother pairs, *C*. *albicans* was detected in either child or mother, or not detected in both samples. Among the 12 child-mother pairs that both carried *C*. *albicans* in the oral cavity, the REAG-B analysis showed 11 identical pairs (61.1%), 0 related pairs, and 1 different pair. Fig 1A demonstrates the REAG-B pattern in 2 representative child-mother pairs. Relatedness analysis was performed by BioNumerics software (Applied Maths, Austin, TX). The *C*. *albicans* isolated from the child-1 and mother-1 were genetically identical, the *C*. *albicans* isolated from the child-5 and mother-5 were genetically different. The *C*. *albicans* from different resources (saliva/oral-rinse, plaque and swab) of the same subject were also analyzed using REAG-B. In one S-ECC children’s mother, the *C*. *albicans* isolated from the saliva showed different pattern from that isolated from the plaque using REAG-B, and was confirmed as two different strains by BioNumerics software. Among the other study subjects, only one type of *C*. *albicans* strain was identified across from saliva/oral-rinse, plaque and swab samples. Regarding caries free children-mother pairs, only one pair of child and mother both had *C*. *albicans* in their samples, REAG-B results indicated that strains from child and mother were identical.

**Fig 2:**
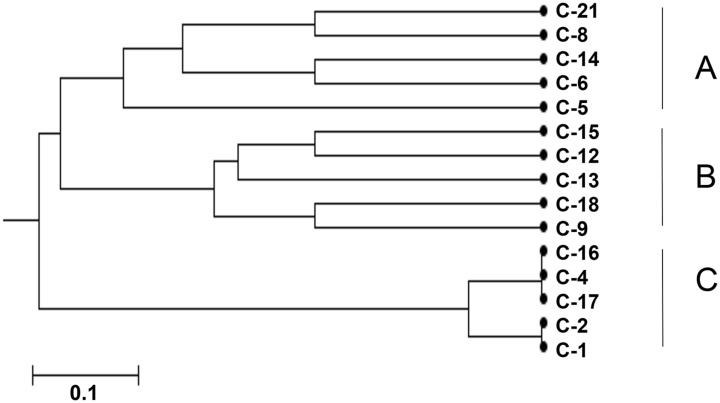
Dendrogram of genetic relationship between 15 *C*. *albicans* isolates from the S-ECC children saliva sample, based on the seven housekeeping loci. *The dendrogram was constructed by using the unweighed pair-group method. The linkage distance is indicated at the bottom.

*C*. *albicans* Isolates from 15 S-ECC children were analyzed with the consensus *C*. *albicans* MLST scheme [49]. New allelic profiles and DSTs were identified in 4 isolates. Of the seven loci investigated for each isolate, four new VPS13 alleles were identified. A UPGMA dendrogram depicting the *C*. *albicans* MLST clustering information between the 15 isolates from ECC children was shown in Fig 2 and were grouped in three main clusters. No enrichment of *C*. *albicans* MLST clusters was found regards to the geographic zip code and caries severity based on caries score in the study samples.

### Susceptibility to caspofungin

**Fig 3:**
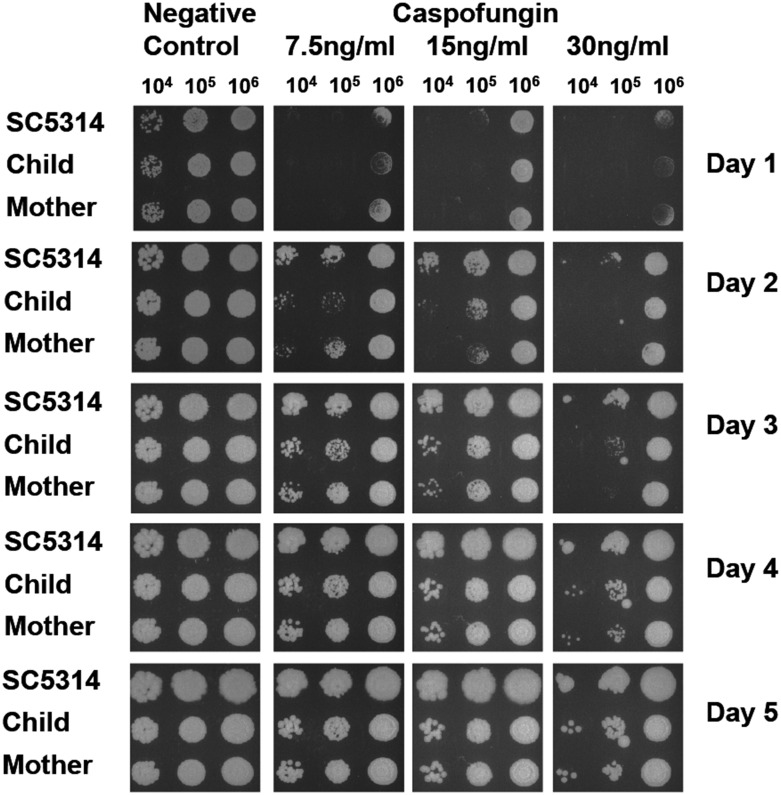
Examples of susceptibility of the clinical isolated *C*. *albicans* from S-ECC children and their mothers to antifungal agents casopfungin compared to *C*. *albicans* wild strain SC5314. From left to right, 10^4^, 10^5^, and 10^6^ cells were spotted on each plate and incubated at 37°C for up to 5 days. Clinical isolated *C*. *albicans* demonstrated less growth and smaller colonies than SC5314 on the YPD medium with caspofungin. However, no significant growth difference was seen between the *C*. *albicans* from S-ECC children and their mothers on the YPD medium with caspofungin.

Clinical strains isolated from 12 pairs S-ECC child-mother were used to perform the susceptibility test to caspofungin to assess microbiological differences among the isolates. In 8 (66.6%) child-mother pair, the strains demonstrated similar susceptible to caspofungin. In 2 child-mother pair, the strains from child were seen forming colonies 1 day earlier on the plate than the strain from the mother. In another 2 child-mother pair, the strains from the child were more susceptible to caspofungin. Examples of susceptibility of the clinical isolated *C*. *albicans* from S-ECC child-mother pair to antifungal agents casopfungin compared to *C*. *albicans* wild strain SC5314 was shown in Fig 3. From left to right, 10^4^, 10^5^, and 10^6^ cells were spotted on each plate and incubated at 37°C for up to 5 days. Clinical isolated *C*. *albicans* demonstrated less growth and smaller colonies than SC5314 on the YPD medium with caspofungin. However, no significant growth difference was seen between the *C*. *albicans* from S-ECC children and their mother on the YPD medium with 7.5 ng/ml and 15 ng/ml of caspofungin.

## Discussion

In the past decade, the fungus *Candida albicans* has been detected more frequently in the oral cavity of children with ECC than caries-free (CF) children [15–21]. For example, high detection rate of *C*. *albicans* was found in plaque of ECC (60–62% vs 14–22% in CF-children [16, 17]) as well as in saliva (67% vs 2% in CF children [18]). Compared to the published literature, we detected 77% in the saliva, 83% in the plaque samples and 44% in swab sample, reinforcing the potential role of *C*. *albicans* in ECC pathogenesis. However, how the children acquire *C*. *albicans* remain an intriguing question. Our findings provide initial evidence that maternal transmission could be at least one source for the *C*. *albicans* infection in S-ECC children. Results from this study reveal that mothers of the children affected by S-ECC also have high *C*. *albicans* carriage (>80% detection in both saliva and plaque samples), and more than 60% of the S-ECC children were carrying the same *C*. *albicans* strains as their mothers. Both findings are novel and have not been reported previously, which provides the framework for larger and longitudinal studies to further validate and expand our observations.

Another important finding from our study is the strong correlation between the salivary and plaque viable cells of *S*. *mutans* and *C*. *albicans*. In the oral cavity, the co-adhesion between *C*. *albicans* and oral bacteria is crucial for *C*. *albicans* colonization and persistence [53]. When sucrose is available, *C*. *albicans* could interact and develop biofilms with *S*. *mutans*, which greatly enhanced the colonization by these organisms on apatitic surfaces [22, 23]. A major mechanism involved in this cross-kingdom association appears to be linked with the *S*. *mutans*–derived exoenzyme glucosyltransferase B (GtfB), a key exopolysacharides (EPS) producer. GtfB can bind to the surface of *C*. *albicans* cells in an active form, produce EPS locally that provide enhanced binding sites for *S*. *mutans*, which in turn promote the formation of biofilms containing elevated EPS amounts and high numbers of *S*. *mutans* and *C*. *albicans* [22, 23, 26]. Once together within a diffusion-limiting EPS-rich matrix, it is conceivable that co-existence of *S*. *mutans* and *C*. *albicans* could induce each other’s growth [23–25] and further enhance acid production in the biofilm (as the fungus is highly acidogenic and aciduric [54, 55]), leading to rampant caries in an animal model of the disease [23].

Our clinical findings are consistent with previous evidence suggesting that *C*. *albicans* and *S*. *mutans* are found together and interact in conditions conducive of ECC. We also found a significant correlation between plaque *C*. *albicans* counts and caries severity (*dmft/dmfs)*. Furthermore, the number of decayed teeth/surfaces in the subjects with both *C*. *albicans* and *S*. *mutans* carriage were clearly higher than that of subjects detected with *S*. *mutans* only, suggesting that the fungal presence might escalate the severity of ECC. It is important to note that plaque sample was collected from the whole dentition combining the plaque from sound and carious tooth surfaces. It is possible that a stronger correlation might have been observed within the plaque from carious lesion teeth. Also, we used colony morphology to distinguish *S*. *mutans* from other streptococci that could also grow in the MSB medium, which has some limitations and could over-estimate the carriage of *S*. *mutans*. Thus, site-specific sample collection combined with species-specific molecular probes will be used in future studies. Nevertheless, further larger scale and longitudinal clinical studies is certainly warranted to validate these findings.

It is noteworthy that *C*. *albicans* enhanced co-colonization and proliferation in the oral cavity of ECC patients could be also explained by the ecological plaque hypothesis [56]. In the context of S-ECC, which is characterized by diet and dietary practices rich in fermentable carbohydrates, the acidogenic microorganisms can rapidly acidify the plaque microenvironment. In turn, the acidic pH conditions would favor the growth of acid-tolerant (aciduric) organisms, such as *C*. *albicans* and *S*. *mutans*, providing an ecological advantage compared to many other oral microbes.

Altogether, these results provide the first insights to further understand the *C*. *albicans* transmission route while confirming the coexistence with *S*. *mutans* and caries severity, which may be important for the pathogenesis of ECC. The limitation of this study was its cross-sectional nature and small sample size (albeit sufficient for this study). Future studies will focus on investigating the vertical transmission of *C*. *albicans* in neonates as well as *C*. *albicans* and *S*. *mutans* colonization and their salivary/plaque levels in the ECC children longitudinally.

## Conclusion

Our results show that the child with S-ECC and the mother were both infected with high levels of *C*. *albicans*, and most of the fungal strains were genetically related, suggesting that the mother might be a primary source for *C*. *albicans* acquisition in the oral cavity of children affected with S-ECC. A strong correlation was also identified between salivary and plaque *C*. *albicans* and *S*. *mutans* carriage using the Spearman’s rank test, suggesting a symbiotic relationship between *C*. *albicans* and *S*. *mutans* in the context of ECC.

## Supporting Information

S1 File Social-demographic and medical background survey form. (PDF)
