# Supplementary material for: Candida albicans Carriage in Children with Severe Early Childhood Caries (S-ECC) and Maternal Relatedness
Source: PLoS One. 2016 Oct 14;11(10):e0164242. doi: 10.1371/journal.pone.0164242 (PMC5065202; doi:10.1371/journal.pone.0164242)
Supplement: S1 File — (PDF) [file pone.0164242.s001.pdf]

C. albicans in S-ECC children and their mother – Medical history

Subject ID __________ Date _____________

Name _______________________ Birth date_______________ Sex F M

Race: American Indian/Alaska Native Asian Native Hawaiian or Pacific Islander Black or African American

Caucasian More than one race unkown or unreported

Ethinicity: Hispanic Non-Hispanic

If minor, parents names _____________________________ ***Relation to the study subject*** _____________________________

Home phone _____________ Cell phone _______________ Email address ____

Mailing address ____________________________________ City ___________________ State ________ Zip ___________

MEDICAL HEALTH HISTORY

Do you have or have you had any of the following?
(Please check any that apply)

- ❑ Cancer or tumor
- ❑ Heart ailment or angina
- ❑ Heart murmur, mitral valve prolapse, heart defect
- ❑ Rheumatic fever or rheumatic heart disease
- ❑ Artificial joint or valve
- ❑ High or low blood pressure
- ❑ Pacemaker
- ❑ Tuberculosis or other lung problems
- ❑ Kidney disease
- ❑ Hepatitis or other liver disease
- ❑ Alcoholism
- ❑ Blood transfusion
- ❑ Diabetes
- ❑ Neurologic condition
- ❑ Epilepsy, seizures, or fainting spells
- ❑ Emotional condition
- ❑ Arthritis
- ❑ Herpes or cold sores
- ❑ AIDS or HIV positive
- ❑ Migraine headaches or frequent headaches
- ❑ Anemia or blood disorders
- ❑ Abnormal bleeding after extractions, surgery, or trauma
- ❑ Hayfever or sinus trouble
- ❑ Allergies or hives
- ❑ Asthma

Do you smoke or use chewing tobacco? ❑ yes ❑ no

Are you allergic to, or have you reacted adversely to any of the following?

- ❑ Latex materials
- ❑ Penicillin or other antibiotics
- ❑ Local anesthetics ("Novocain")
- ❑ Codeine or other narcotics
- ❑ Sulfa drugs
- ❑ Barbiturates, sedatives, or sleeping pills
- ❑ Aspirin
- ❑ Other:_____________________________________

Are you taking any of the following?

- ❑ Aspirin
- ❑ Anticoagulants (blood thinners)
- ❑ Antibiotics or sulfa drugs
- ❑ High blood pressure medicine
- ❑ Antidepressants or tranquilizers
- ❑ Insulin, Orinase, or other diabetes drug
- ❑ Nitroglycerin
- ❑ Cortisone or other steroids
- ❑ Osteoporosis (bone density) medicine
- ❑ Other:_____________________________________
  ____

Women:

- ❑ May be pregnant
  Expected delivery date: _____________
- ❑ Taking hormones or contraceptives

C. albicans in S-ECC children and their mother – Medical history

Subject ID __________ Date _____________

Please answer the following study related questions, thank you!

1. *Do you (your child) have history of yeast infection?*
- ❑ Athlete's foot
- ❑ Ringworn
- ❑ Cradle cap (children)
- ❑ Oral thrush
- ❑ Denture-related stomatitis
- ❑ Agular stomatitis
- ❑ systematic candidiasis
- ❑ other: ______________

2. *Have you (your child) had long term (>3month) antibiotics use?*
- ❑ No
- ❑ Yes, please specify ____________________

3. *Have you (your child) had antifungal therapy in the past 3 month?*
- ❑ No
- ❑ Yes, please specify ___________________

4. *Were you (your child) a low birth weight infant?*
- ❑ No
- ❑ Yes, the birth weight was ___________

5. *How often you (your child) brush teeth?*
- ❑ Twice/daily
- ❑ Once/daily
- ❑ Not everyday
- ❑ Neve

6. *Who is your child's direct care provider at home?*
- ❑ mom
- ❑ dad
- ❑ grandmother
- ❑ grandfather
- ❑ others___________

7. *Does your child attend daycare center?*
- ❑ Yes, part time
- ❑ Yes, full time
- ❑ No
